# Death or survival, which you measure may affect conclusions: A methodological study

**DOI:** 10.1002/hsr2.905

**Published:** 2022-10-25

**Authors:** Jake Shannin, Babette A. Brumback

**Affiliations:** ^1^ Department of Statistics University of Florida Gainesville Florida USA; ^2^ Department of Biostatistics University of Florida Gainesville Florida USA

**Keywords:** effect‐measure modification, odds ratio, relative risks, risk difference, vaccine efficacy

## Abstract

**Background and Aims:**

Considering the opposite outcome—for example, survival instead of death—may affect conclusions about which subpopulation benefits more from a treatment or suffers more from an exposure.

**Methods:**

For case studies on death following COVID‐19 and bankruptcy following melanoma, we compute and interpret the relative risk, odds ratio, and risk difference for different age groups. Since there is no established effect measure or outcome for either study, we redo these analyses for survival and solvency.

**Results:**

In a case study on COVID‐19 that ignores confounding, the relative risk of death suggested that 40–49‐year‐old Mexicans with COVID‐19 suffered more from their unprepared healthcare system, using Italy's system as a baseline, than their 60–69‐year‐old counterparts. The relative risk of survival and the risk difference suggested the opposite conclusion. A similar phenomenon occurred in a case study on bankruptcy following melanoma treatment.

**Conclusion:**

To increase transparency around this paradox, researchers reporting one outcome should note if considering the opposite outcome would yield different conclusions. When possible, researchers should also report or estimate underlying risks alongside effect measures.

## 1 INTRODUCTION

Researchers may think it arbitrary whether they measure survival or mortality. Unfortunately, this choice may affect conclusions about how a treatment or exposure differently affects different subpopulations. This paper looks at real‐world examples of this phenomenon in death following COVID‐19 and bankruptcy following melanoma treatment. Whether this phenomenon occurs depends on choice of effect measure: The risk difference (RD) $\left(p2‐p1\right)$ and odds ratio (OR) $\left(\frac{p2\left(1-p1\right)}{p1\left(1-p2\right)}\right)$ are immune from this phenomenon, but the relative risk (RR) $\left(\frac{p2}{p1}\right)$ and the cumulative hazard ratio (HR) $\left(\frac{\log\left(1‐p2\right)}{\log\left(1‐p1\right)}\right)$ are not. From patterns in the case studies, we prove a theorem: If the relative risks of survival and death agree as to which subpopulation benefits most from a treatment, then the cumulative hazard ratios, the RD, and the OR will agree with them.

Prior research, including Dr. Mindel Sheps' *Shall We Count the Living or the Dead?* (1958),^3^ discusses this phenomenon. In a field trial of a poliomyelitis vaccine, Sheps recommends comparing treatment groups via vaccine efficacy, an effect measure closely associated with the relative risk of contracting poliomyelitis. As in our COVID‐19 case study (Section 2), we believe that the RD may be a more appropriate effect measure, especially if vaccines are scarce. Furthermore, if risks of poliomyelitis are low, then the RD and the relative risk for the opposite outcome (not contracting poliomyelitis) will yield similar conclusions about which subpopulation benefits more from vaccination (Section 4.1).

In a study where smoking increases the risk of lung cancer, Sheps suggests filtering out patients who die from causes other than lung cancer and then considering the relative risk of not developing and dying from lung cancer. Her nuanced analysis establishes that there is no universal procedure for choosing an effect measure or outcome, though several have tried for both. Huitfeldt et al.^1^ suggest the Switch RR, equal to RR – 1 if p2<p1 or otherwise RD/(1−p1), though it may mimic a relative risk in cases where the RD may be more prudent, such as the poliomyelitis vaccine trial. Rothman et al.^2^ imply a convention for choosing between opposite outcomes: “Sheps^3^ once asked, ‘Shall we count the living or the dead?’ Death is an event, but survival is not. Hence, to use the sufficient‐component cause model, we must count the dead.” However, there may be cases where opposite outcomes are both events, such as sleeping <8 h and sleeping ≥8 h. Consider a sufficient‐component cause model in which insomnia and early high school start times are both sufficient causes for sleeping <8 h. Lack of insomnia and later high school start times are necessary but insufficient causes for sleeping ≥8 h, so a sufficient‐component cause model for sleeping ≥8 h is not immediate.

Let p1 and p2 denote the proportion of participants in groups 1 and 2 reaching the measured outcome. When risks (p1,p2) are reported or estimated alongside effect measures, evaluating choices of outcome and effect measure is straightforward. However, risks are often omitted and occasionally incalculable. Of 222 papers studied by Schwartz et al.,^4^ 68% failed to report or estimate risks in the abstract, 35% failed to report risks anywhere, and 13% failed to make risks calculable. We urge researchers to report or estimate risks whenever possible.

The other relative risk, $\mathrm{RR}*\;=\frac{1‐p1}{1‐p2}$, represents the RR for the opposite outcome, allowing us to analyze the joint impact of choice of effect measure and choice of outcome. We similarly define the other cumulative hazard ratio, $\mathrm{HR}*\;=\frac{\log\left(p1\right)}{\log\left(p2\right)}$, to represent HR for the opposite outcome. While the hazard ratio depends on time, the cumulative hazard ratio equals the hazard ratio at all times if the proportional hazards assumption holds.

This paper is organized as follows:

- Ignoring confounding for ease of exposition, Section 2 compares choices of outcome and effect measure in analyzing how age modifies the effect of healthcare system on risk of death from COVID‐19.
- Section 3 compares effect measure‐outcome combinations in a study on how melanoma and its treatment differently increase different age groups' risks of bankruptcy.
- Section 4 discusses patterns in these two case studies and formalizes them into a theorem proved in the Supporting Information.

## 2 CASE STUDY: COVID‐19 MORTALITY, AGE, AND COUNTRY OF TREATMENT

### 2.1 Background

The risk of death or survival in patients with COVID‐19 depends heavily on many factors including their age^5^ and the relative prevalence of COVID‐19 in their healthcare system, relative to that system's capacity. In this case study, we use different effect measures to investigate how the age of patients with COVID‐19 modifies the effect their healthcare system has on their risk of death or survival.

For purposes of this case study, we will neglect confounders, that is, mutual causes of COVID‐19 mortality and relative prevalence. Such variables include the rate of testing: Increased testing decreases the measured death rate of COVID‐19 by revealing asymptomatic and weakly symptomatic cases.^6^ Increased testing also decreases the relative prevalence of COVID‐19, since countries with increased testing generally detect COVID‐19 outbreaks in time to implement appropriate policy actions to prevent the outbreak from overwhelming their healthcare systems.^7^ Thus, increased testing partially “explains away” the strong association between COVID‐19 mortality and relative prevalence. Other potential confounders include genetics and lifestyle. We, therefore, intend this case study as an illustrative example of how considering different effect measures, or survival instead of death, may affect conclusions about which subpopulation suffers more from an exposure.

### 2.2 Effect measures and outcomes

Younger people generally have less risk of dying from COVID‐19. Italian 40–49‐year‐olds are no exception with a death rate of p1=0.9% as of June 3, 2020.^8^ Mexican 40–49‐year‐olds are not as fortunate with a death rate of p2=7.5% (RR = 8.33) as of June 3, 2020.^9^ Among 60–69‐year‐olds, Italians and Mexicans have respective Case Fatality Rates of p3=10.6% and p4=25.3% (RR = 2.39). While COVID‐19 overwhelmed both countries' healthcare systems, it caught Mexico particularly unprepared,^10^ at least partially explaining these disparate death rates. Other explanatory variables include Mexico's increased absolute prevalence and accelerated onset of pre‐existing conditions that increase the risk of death from COVID‐19.^10^ We will look at how age modifies each of our effect measures.

The relative risk (RR) and the odds ratio (OR) find the disparity between Mexican and Italian 40–49‐year‐olds more alarming than that disparity among 60–69‐year‐olds.

- RR: A 40–49‐year‐old person from Mexico with COVID‐19 is 8.33 times as likely to die as a 40–49‐year‐old person from Italy with COVID‐19. In contrast, a 60–69‐year‐old person from Mexico with COVID‐19 is only 2.39 times as likely to die as their Italian counterpart.
- OR: The odds of Mexican 40–49‐year‐olds with COVID‐19 dying are 8.93 times those of Italian 40–49‐year‐olds. In contrast, the odds of Mexican 60–69‐year‐olds with COVID‐19 dying are only 2.86 times those of their Italian counterparts.

These effect measures may lead stakeholders to conclude that countries with underprepared healthcare systems should focus on middle‐aged patients whose deaths are possible but typically preventable, rather than on older patients who have a substantial chance of dying even if prioritized for treatment.

The other relative risk (RR*), which corresponds to the relative risk for survival, and the risk difference (RD) yield the opposite conclusion.

- RR*: A 60–69‐year‐old person from Italy with COVID‐19 is 19.7% (RR* = 1.197) more likely to survive infection than a 60–69‐year‐old person from Mexico with COVID‐19. In contrast, a 40–49‐year‐old person from Italy with COVID‐19 is just 7.1% (RR* = 1.071) more likely to survive infection than a 40–49‐year‐old person from Mexico with COVID‐19.
- RD: The risk of death among 60–69‐year‐old Mexicans with COVID‐19 is 0.147 higher than the risk of death among 60–69‐year‐old Italians with COVID‐19. In contrast, the risk of death among 40–49‐year‐old Mexicans with COVID‐19 is only 0.066 higher than that risk among their Italian counterparts.
- In terms of the number needed to treat (1/RD), and using our assumption of causation, if 6.8 (1/0.147) 60–69‐year‐old Mexicans with COVID‐19 were instead treated under Italy's healthcare system, we would expect 1 fewer death. In contrast, we would have to treat an estimated 15.2 40–49‐year‐old Mexicans with COVID‐19 under Italy's healthcare system to save 1 life.

The RD is typically the effect measure most suitable for identifying which subpopulation would benefit the most from treatment.^11^, ^12^, ^13^

### 2.3 Discussion

The COVID‐19 pandemic caught healthcare systems unprepared, requiring them to choose which subpopulations to treat with limited resources. Data detailing these subpopulations' risks of death or survival from COVID‐19 with and without treatment inform such decisions. Our case study suggests that which outcome (death or survival) is used, along with the effect measure used, may determine this decision: Mexico may target treatment toward 40–49‐year‐old patients with COVID‐19 if health officials base their decision on the RR for death, OR, or either HR; alternatively, Mexico may prioritize treating 60–69‐year‐old patients with COVID‐19 after comparing age‐nationality groups with the RD or the relative risk of surviving COVID‐19 (RR*). The effect measures we studied differed between strata substantially (RR: 8.33 vs. 2.39; RD: 0.066 vs. 0.147), showing that conclusions may vary egregiously across opposite outcomes and different effect measures.

## 3 CASE STUDY: YOUNG AND OLD MELANOMA PATIENTS' RISKS OF BANKRUPTCY

### 3.1 Background

Patients with melanoma are on average HR = 2.08 times as likely to file for bankruptcy at any given moment as their matched controls. Ramsey et al.^14^ found that p1 = 0.00830 of 20–34‐year‐old patients with melanoma, but only p2 = 0.00384 of their matched controls, filed for bankruptcy during an average year of their study. In contrast, only p3 = 0.00140 of 80–90‐year‐old patients with melanoma, and only p4 = 0.00045 of their matched controls, filed for bankruptcy during an average year. Assuming that differences are statistically significant effects of melanoma, we will look at how age modifies measuring the effect melanoma has on risk of bankruptcy, or oppositely, on risk of avoiding bankruptcy.

### 3.2 Effect measures and outcomes

The RR and the OR suggest that melanoma more sharply increases 80–89‐year‐old patients' risk of bankruptcy.

- RR: 80–89‐year‐old patients with melanoma are 3.11 times as likely to file for bankruptcy as their matched controls. In contrast, 20–34‐year‐old patients with melanoma are only 2.16 times as likely to file for bankruptcy as their matched controls.
- OR: The odds of 80–89‐year‐old patients with melanoma filing for bankruptcy are 3.11 times those of their matched controls. In contrast, the odds of 20–34‐year‐old patients with melanoma filing for bankruptcy are only 2.17 times those of their matched controls.

The relative risk of the opposite outcome (RR*), avoiding bankruptcy, and the RD suggest the opposite conclusion.

- RR*: A matched control is 0.45% (RR* = 1.0045) more likely to avoid bankruptcy than a 20–34‐year‐old patient with melanoma. In contrast, a matched control is only 0.095% (RR* = 1.00095) more likely to avoid bankruptcy than an 80–89‐year‐old patient with melanoma.
- RD: The risk of bankruptcy among 20–34‐year‐old patients with melanoma is 0.00446 higher than the risk of bankruptcy among their matched controls. In contrast, the risk of bankruptcy among 80–89‐year‐old patients with melanoma is only 0.00095 higher than the risk of bankruptcy among their matched controls.
- In terms of the number needed to treat (1/RD), if we relieved 224 20–34‐year‐old patients with melanoma of its financial effects, we would expect 1 fewer bankruptcy. In contrast, we would have to relieve an estimated 1053 80–89‐year‐old patients with melanoma from its financial burden to prevent 1 bankruptcy.

### 3.3 Discussion

Hospitals often face difficult decisions to stay financially solvent while ensuring that their patients get the care they need. Governments benefit from an understanding of how medical expenses affect citizens' financial stability since they choose which populations to target with interventions such as Medicare. In causal contexts, effect‐measure modification is the study of how a modifier affects the extent to which an exposure causes a disease. In this case study, the modifier is age, the exposure is melanoma, and the disease is bankruptcy. The direction in which age modifies the effect of melanoma on bankruptcy depends on effect measure and, when using relative risks, contradicts the direction in which age modifies melanoma's effect on avoiding bankruptcy. Researchers in health science and economics alike should provide risks when possible and note if their conclusions vary between effect measures or opposite outcomes.

## 4 PATTERNS ACROSS CASE STUDIES AND CORRESPONDING THEORETICAL RESULTS

Researchers often choose between opposite outcomes, such as survival and death. The RD and the OR are unaffected by this choice, but the RR and the HR may suggest opposite conclusions. Moreover, if the relative risk for one outcome and the relative risk for the other outcome suggest effect‐measure modification in the same direction, then so will all other aforementioned effect measures for either outcome (Section 4.2).

### 4.1 Similarities between COVID‐19 and melanoma case studies

In our age‐stratified case studies on COVID‐19 treatment in Italy and Mexico (Section 2) and bankruptcy following melanoma treatment (Section 3), we reached contradictory qualitative conclusions by considering the relative risks of death and survival, or bankruptcy and solvency. In both case studies, the RD suggested the same conclusion as the relative risk for the more common outcome (survival or solvency), while the OR sided with the relative risk for the rarer outcome (death or bankruptcy). When the risks of death or bankruptcy were small, we observed RR ≈ OR and 1 + RD ≈ RR*, approximations which Taylor expansions show are true in general.^1^

### 4.2 Theorem (proof in Supporting Information)

These similarities turn out not to be mere coincidences resulting from our small sample size (*n* = 2 case studies). In the Supporting Information, we prove the following theorem: (i) For any risks p1, p2, p3, and p4 between 0 and 1, if the two relative risks RR and RR* agree (about which subpopulation benefitted more from the treatment), then so does the entire set of our effect measures {RR, RR*, HR, HR*, RD, OR}. (ii) Furthermore, if risks p1, p2, p3, and p4 are randomly sampled from the uniform (0,1) distribution, then the probability of {RR, RR*, HR, HR*, RD, OR} all agreeing is 5/6.

Section A of the Supporting Information proves (i) algebraically. Section B of the Supporting Information proves (ii) using the intermediate value theorem.

### 4.3 Large and small effect measures

When two effect measure‐outcome combinations agree about which subpopulation benefitted more from treatment, they may incite different impressions about how much more one subpopulation benefitted than the other. This may follow from differing impressions within a subpopulation. Alternatively, this may occur when two effect measure‐outcome combinations *nearly* disagree, that is, a small change to the underlying risks would lead the effect measures to disagree about which subpopulation benefitted more from treatment.

#### 4.3.1 Differing impressions within a subpopulation

While any two effect measure‐outcome combinations are sure to agree about whether a single subpopulation benefitted from a treatment, they may incite different impressions about the magnitude of benefit. In Baden et al.'s^15^ phase 3 trial, 11 of 14134 (p2=0.078%) patients receiving the mRNA‐1273 vaccine series per‐protocol contracted symptomatic COVID‐19, compared to 185 of 14073 (p1=1.315%) patients receiving a placebo series per‐protocol. The vaccine efficacy (1 – RR = 94.1%) clearly communicates the vaccine's protection, whereas the RD (–0.0124) varies with the prevalence of COVID‐19 and does not make headlines.

#### 4.3.2 Near disagreement

One effect measure‐outcome combination may suggest that one subpopulation responded substantially more to a treatment, while another may suggest a milder difference in response. In Baden et al.'s^14^ trial, 4 of 3206 (p2=0.12%) patients at risk for severe COVID‐19 receiving the mRNA‐1273 vaccine series per‐protocol contracted symptomatic COVID‐19, compared to 43 of 3167 (p1=1.36%) at‐risk patients receiving a placebo series per‐protocol. This gives a vaccine efficacy of 1 – RR = 90.8% and a risk difference of RD = –0.0123. Similarly, 7 of 10928 (p4=0.06%) not‐at‐risk patients receiving the vaccine contracted symptomatic COVID‐19, compared to 142 of 10906 (p3=1.30%) not‐at‐risk patients receiving a placebo. The vaccine efficacy, 90.8% for at‐risk patients and 95.1% for not‐at‐risk patients, and the RD, –0.0123 for at‐risk patients and –0.0124 for not‐at‐risk patients, agree (ignoring statistical significance) that patients not at risk for severe COVID‐19 responded more strongly to the vaccine. However, this agreement is not substantial: Had 1 more not‐ask‐risk patient receiving the vaccine contracted symptomatic COVID‐19, the two effect measures would disagree.

#### 4.3.3 Implications

Our theorem may allow researchers to conclude agreement between effect measure‐outcome combinations without computing each of them. For example, if RR and RR* agree, then RR and RD automatically agree. However, the potential for near disagreement warns that this agreement may not be substantial. Therefore, we urge researchers to consider and report risks or multiple effect measures when possible.

### 4.4 How to apply

Our findings are of interest to researchers choosing between effect measures and opposite outcomes and to researchers performing meta‐analyses over literature employing varying effect measures and outcome codifications. Our strongest recommendation is that researchers report risks whenever possible. Since no single effect measure or outcome is uniformly superior, we suggest researchers report multiple effect measures, such as the two relative risks, unless there is a standard or purpose‐informed choice.

In some fields, there is a standard effect measure‐outcome combination. For example, HIV trials defining participants with less than 50 viral RNA copies per milliliter as having reached the measured outcome typically use RD, while trials defining virologic failure as the measured outcome typically use RR or HR.^16^ In some studies, the purpose of the study informs the effect measure‐outcome choice. For instance, a study recommending a population for prioritized COVID‐19 vaccination may employ the RD to save the most lives.

In contexts where there is no clear choice, we recommend that researchers report both relative risks. If they suggest the same conclusion, then our theorem shows that the studied factor also modifies HR, HR*, RD, and OR in the same direction. For example, a meta‐analysis of studies testing for RD modification could include a study that showed relative risk modification for each of two opposite outcomes.

#### 4.4.1 Bivariate delta method

Brumback and Berg^17^ suggested the multivariate delta method to test the alternative hypothesis that a factor modifies the RR, RD, and OR in the same direction. This method involves considering a joint distribution with a dimension for each of the three effect measures. We improve on this recommendation, increasing the strength of the alternative hypothesis and reducing the dimensionality of the applicable joint distribution: We suggest using the bivariate delta method to test the alternative hypothesis that a factor modifies RR, RR*, HR, HR*, RD, and OR in the same direction. By our theorem, it suffices to consider the joint distribution of just the two relative risk ratios $\frac{p2p3}{p1p4}\;\text{and}\;\frac{\left(1-p1\right)\left(1-p4\right)}{\left(1-p2\right)\left(1-p3\right)}$. We reject the null hypothesis if the 100(1 – *α*)% simultaneous confidence region for the relative risk ratios lies completely within the (>1,>1) region or the (<1,<1) region.

### 4.5 Future research

- Gilbert et al.^18^ adapt survivor average causal effect (SACE) analysis to principal surrogate (PS) analysis on the HVTN 505 HIV‐1 vaccine trial. Their analysis found qualitative vaccine efficacy modification by a post‐randomization biomarker. Future research may adapt SACE to PS in the context of effect measures besides vaccine efficacy (which corresponds to the relative risk). Furthering our consideration of the opposite outcome, future research could formulate the “other” survivor average causal effect (SACE*) to be the average causal effect in participants who would be non‐survivors (e.g., who would experience HIV infection) regardless of assignment to the control or treatment group.
- Since the two relative risks correspond to Cheng's preventative and generative causal powers, existing research^19^ relating the causal powers to Bayesian networks could be readily extended to the two relative risks.
- Huitfeldt et al.^20^ show confounding and monotonicity assumptions for reaching counterfactual interpretations of the two relative risks and their reciprocals. Further research could, given those assumptions, assess the possibility and frequency of disagreement between these counterfactual outcome state transition (COST) parameters and non‐COST effect measures (HR, HR*, RD, OR).

## AUTHOR CONTRIBUTIONS

**Jake Shannin**: Conceptualization; Formal analysis; Investigation; Methodology; Project administration; Software; Validation; Writing–original draft; Writing–review & editing. **Babette A Brumback**: Conceptualization; Formal analysis; Methodology; Project administration; Supervision; Validation; Writing–review & editing.

## CONFLICT OF INTEREST

The authors declare no conflict of interest.

## TRANSPARENCY STATEMENT

The lead author Jake Shannin affirms that this manuscript is an honest, accurate, and transparent account of the study being reported; that no important aspects of the study have been omitted; and that any discrepancies from the study as planned (and, if relevant, registered) have been explained.

## Supporting information

Supplementary information.

Supplementary information.

## Data Availability

The authors confirm that the data supporting the findings of this study are available within the article and its Supporting Information.
