# Supplementary material for: Death or survival, which you measure may affect conclusions: A methodological study
Source: Health Sci Rep. 2022 Oct 25;5(6):e905. doi: 10.1002/hsr2.905 (PMC9596942; doi:10.1002/hsr2.905)
Supplement: Supplementary file 1 — Supplementary information. [file HSR2-5-e905-s002.pdf]

Supplemental Materials: Death or Survival, Which You Measure May Affect Conclusions: A Methodological Study

Jake Shannin and Babette A. Brumback, Ph.D.

September 2022

A Supplement: If Both Relative Risks Agree, then so do Both Hazard Ratios, the Risk Difference, and the Odds Ratio.

In this supplement, we show that the relative risks agree if and only if all six effect measures agree. We can prove this in two ways: algebraically and analytically. In this section, we provide the algebraic proof. The analytic proof, partially presented in Supplement B, is more technical than the algebraic proof but offers a framework useful for future proofs, such as our proof in Supplement B that all six effect measures agree with probability $\frac{5}{6}$ when risks are randomly sampled from the uniform (0,1) distribution.

To ensure all effect measures are defined, we restrict the risks $p1, p2, p3$, and $p4$ to the open unit interval. If at least two risks are 0 or at least two risks are 1, the presence and direction of effect modification are clear without the use of effect measures. If exactly one risk is 0 and at most one risk is 1, or vice versa, then it is feasible to apply our theorem by defining otherwise-undefined effect measures using appropriate one-sided limits. For example, if $p1 = 0$ and $p2 = 0.3$, then we may define RR $= \lim_{p1 \to 0^+} \frac{0.3}{p1} =$ OR $=$ HR $=$ HR$^* = \infty$.

A.1 Qualitative Effect Modification: If either $p1 < p2$ or $p3 < p4$ but not both, then all six effect measures agree.

Relabelling strata as necessary, suppose that $p1 < p2$ and $p3 \geq p4$. Let $\mathrm{RR_P}$ denote the relative risk for the $(p1, p2)$ stratum, and let $\mathrm{RR_Q}$ denote the relative risk for the $(p3, p4)$ stratum. We similarly define this subscript notation for other effect measures. Then

- $\mathrm{RR}_P > 1$ and $\mathrm{RR}_Q \leq 1$,
- $\mathrm{RR^*}_P > 1$ and $\mathrm{RR^*}_Q \leq 1$,
- $\mathrm{HR}_P > 1$ and $\mathrm{HR}_Q \leq 1$,
- $\mathrm{HR^*}_P > 1$ and $\mathrm{HR^*}_Q \leq 1$,
- $\mathrm{RD}_P > 0$ and $\mathrm{RD}_Q \leq 0$, and
- $\mathrm{OR}_P > 1$ and $\mathrm{OR}_Q \leq 1$.

Hence all six effect measures agree as desired.

A.2 If RR and RR* agree, then HR* agrees with them.

If $p1 < p2$ or $p3 < p4$ but not both, we have qualitative effect modification, so by Section A.1, {RR, RR*, HR*} agrees as desired. Otherwise, we can relabel treatment groups and strata as necessary so that $1 < \frac{p2}{p1} < \frac{p4}{p3}$. Suppose that RR and RR* agree, giving that $1 < \frac{1-p1}{1-p2} < \frac{1-p3}{1-p4}$. We will show that $\frac{\log p1}{\log p2} < \frac{\log p3}{\log p4}$ in the two below cases. Note that we write each case as a standalone proposition; i.e., we rewrite aforementioned suppositions that we use in each case's proof.

Suppose $p4 > p2 > p1$ and $p4 > p3 > p1$. Then RR and HR* agree. Taking the logarithm of both side of $\frac{p2}{p1} < \frac{p4}{p3}$ gives that $\log p2 - \log p1 < \log p4 - \log p3$, from which it follows that $\frac{\log p1}{\log p2} < \frac{\frac{\log p3}{\log p4} - 1}{\frac{\log p2}{\log p4}} + 1$. And since $p4 > p2$, we have $\frac{\frac{\log p3}{\log p4} - 1}{\frac{\log p2}{\log p4}} + 1 < \frac{\log p3}{\log p4}$. Transitively, $\frac{\log p1}{\log p2} < \frac{\log p3}{\log p4}$, so RR and

HR* agree as desired.

Suppose $p4 < p2 < 1 < \frac{1-p1}{1-p2} < \frac{1-p3}{1-p4}$. Then RR* and HR* agree.

For $i \in \{1,2,3,4\}$, let $\tilde{p}_i = 1 - p_i$. Then

$$\frac{\log p1}{\log p2} = \frac{\log(1-\tilde{p}_2 \text{RR*}_P)}{\log(1-\tilde{p}_2)} < \frac{\log(1-\tilde{p}_4 \text{RR*}_P)}{\log(1-\tilde{p}_4)} < \frac{\log(1-\tilde{p}_4 \text{RR*}_Q)}{\log(1-\tilde{p}_4)} = \frac{\log p3}{\log p4} \tag{1}$$

with inequalities justified in Supplement B. Since $\text{RR*}_\text{P} < \text{RR*}_\text{Q}$ and $\text{HR*}_\text{P} < \text{HR*}_\text{Q}$, we have that RR* and HR* agree.

In the remaining case that $p2 = p4$, all six effect measures agree, including RR, RR*, and HR*. Hence if the two relative risks agree, then the other cumulative hazard ratio agrees with them as desired.

A.3 If RR and RR* agree, then HR agrees with them.

Conceptually: The relative risks are concordant with each other for the opposite outcome, so if RR and RR* agree for one outcome, they also agree for the opposite outcome. By the previous subsection, this implies that HR* for the opposite outcome agrees. But HR* for the opposite outcome is concordant with HR, so if RR and RR* agree, then HR agrees with them as desired.

Algebraically: We showed in Section A.2 that for any $w, x, y, z \in (0,1)$, if $\frac{x}{w} > \frac{z}{y}$ and $\frac{1-w}{1-x} > \frac{1-y}{1-z}$, then $\frac{\log w}{\log x} > \frac{\log y}{\log z}$. Let $w = 1-p2, x = 1-p1, y = 1-p4$, and $z = 1-p3$. Then the above gives that if $\frac{1-p1}{1-p2} > \frac{1-p3}{1-p4}$ and $\frac{p2}{p1} > \frac{p4}{p3}$, then $\frac{\log(1-p2)}{\log(1-p1)} > \frac{\log(1-p4)}{\log(1-p3)}$. Similarly, if $\frac{1-p1}{1-p2} < \frac{1-p3}{1-p4}$ and $\frac{p2}{p1} < \frac{p4}{p3}$, then $\frac{\log(1-p2)}{\log(1-p1)} < \frac{\log(1-p4)}{\log(1-p3)}$. Therefore if the two relative risks agree, then the cumulative hazard ratio agrees with them as desired.

A.4 If RR and RR* agree, then RD agrees with them.

We begin by presenting two standalone sufficient conditions for agreement:

If $\text{RR*}_\text{P} < \text{RR*}_\text{Q}$ and $p3 \leq p1$, then RR* and RD agree.

Suppose that $\frac{1-p1}{1-p2} < \frac{1-p3}{1-p4}$. Equivalently, $\frac{p3-p4}{1-p3} < \frac{p1-p2}{1-p1}$. Therefore $p4 - p3 > \frac{(p2-p1)(1-p3)}{1-p1} \geq p2 - p1$ by our assumption that $p3 \leq p1$. Since $\text{RD}_\text{P} < \text{RD}_\text{Q}$,

the other relative risk and the risk difference agree.

If $RR_P < RR_Q$ and $p3 \geq p1$, then RR and RD agree.
Suppose that $\frac{p2}{p1} < \frac{p4}{p3}$. Then $\frac{p2-p1}{p1} < \frac{p4-p3}{p3}$. Since $p3 \geq p1$, we have $p4 - p3 > (p2 - p1)\frac{p3}{p1} \geq p2 - p1$. Hence $RD_P < RD_Q$, so the relative risk and the risk difference agree.

Suppose that RR and RR* agree. If there is qualitative effect modification, all effect measures, including RD, agree. Otherwise, we relabel strata as necessary so that $RR_P < RR_Q$ and $RR^*_P < RR^*_Q$. Then one of the two above results applies, i.e., the risk difference must always agree with one of the relative risks. We conclude that if the two relative risks agree, then the risk difference must agree with them as desired.

A.5 If RR and RR* agree, then OR agrees with them.

This follows from the odds ratio being the product of the two relative risks: Suppose that $\frac{p2}{p1} < \frac{p4}{p3}$ and $\frac{1-p1}{1-p2} < \frac{1-p3}{1-p4}$. Multiplying these inequalities, we get that $\frac{p2(1-p1)}{p1(1-p2)} < \frac{p4(1-p3)}{p3(1-p4)}$. Since $RR_P < RR_Q$ and $RR^*_P < RR^*_Q$ together imply $OR_P < OR_Q$, we conclude that if the two relative risks agree, then the odds ratio agrees with them as desired.

A.6 Conclusion

From the preceding sections, we conclude that if the two relative risks agree, then so must the set of all six of our effect measures. Along the way, we found several sufficient conditions for agreement between effect measures:

- If $p4 > p2 > p1$ and $p4 > p3 > p1$ are both true or both false, then RR and HR* agree. Otherwise, RR* and HR* agree.
- If $p4 < p2$ and $\frac{1-p1}{1-p2} < \frac{1-p3}{1-p4}$ are both true or both false, then RR* and HR* agree. Otherwise, RR and HR* agree.
- If $RR^*_P < RR^*_Q$ and $p3 \leq p1$ are both true or both false, then RR* and RD agree. Otherwise, RR and RD agree.
- If $RR_P < RR_Q$ and $p3 \geq p1$ are both true or both false, then RR and RD agree. Otherwise, RR* and RD agree.

B Supplement: Probability of Agreement

In this supplement, we show that the probability that the two relative risks agree — or equivalently by our theorem in Supplement A, that all our effect measures agree — is $\frac{5}{6}$ when risks are randomly sampled from the (0,1) uniform distribution.

Analytic proof that when RR and RR* agree, RD agrees with them

In Supplement A, we proved algebraically that if the two relative risks agree, then the entire set of our effect measures {RR, RR*, HR, HR*, RD, OR} agrees. In this section, we provide an analytic proof that when the two relative risks agree, the risk difference agrees with them. We will use the framework of this proof in our proof that the probability of such agreement is $\frac{5}{6}$ when risks are randomly sampled from the uniform $(0,1)$ distribution.

As in Supplement A, let population P describe the $(p1, p2)$ stratum, and let population Q describe the $(p3, p4)$ stratum. For each effect measure EM, given risks $p_1, p_2, p_3 \in (0,1)$, we define $p_4^{*\text{EM}}$ to be the *critical* value of p_4 at which the EM effect measure would indicate that the treatment or exposure affects populations P and Q equally. Notably, $p_4^{*\text{RR}} = \frac{p2p3}{p1}$, $p_4^{*\text{RD}} = p2 + p3 - p1$, and $p_4^{*\text{RR}^*} = 1 - \frac{(1-p2)(1-p3)}{1-p1}$.

The importance of $p4^*$ is that two effect measures disagree if (and only if) the true value of $p4$ falls between $p4^*$ for each effect measure. We will show this for $p2 > p1$ and $p4 > p3$. Let EM and FM be any two effect measures. Suppose that $p_4^{*\text{EM}} < p4 < p_4^{*\text{FM}}$. The left inequality gives that the EM effect measure considers population Q to respond to treatment or exposure more strongly than population P, since $p4$ is more than what it would be were there no EM modification. Similarly, the right inequality gives that the FM effect measure considers population P to respond to treatment or exposure more strongly than population Q. Hence EM and FM disagree as to the direction of effect-measure modification.

We start by looking at the four examples in Table 1. For each example, the theorem holds for all $p_4 \in (0,1)$, because p_4^{*RD} is between p_4^{*RR} and $p_4^{*RR^*}$. The planes $p_1 = p_2$ and $p_1 = p_3$ divide $(0,1)^3$, the space of (p_1, p_2, p_3), into four open regions. Let us call each region by the boldface of the example it contains. For example, $(p_1 = 0.7, p_2 = 0.5, p_3 = 0.8)$ is in region **B** as $p_1 > p_2$

Example	$p1$	$p2$	$p3$	$p4^{*RR}$	$p4^{*RD}$	$p4^{*RR^*}$
A	0.1	0.2	0.3	0.6	0.4	0.38
B	0.2	0.1	0.3	0.15	0.2	0.21
C	0.2	0.3	0.1	0.15	0.2	0.21
D	0.3	0.1	0.2	0.67	0	-0.03

Table 1: RR Relative Risk of Death; RD Risk Difference; RR* Reciprocal of Relative Risk of Survival. Suppose the risks $p1, p2,$ and $p3$ are all strictly between 0 and 1. We compute $p4^{*RR}$, $p4^{*RD}$, and $p4^{*RR^*}$ such that there is no effect-measure modification on the RR, RD, and RR* scales, respectively. We consider a representative example (A, B, C, D) for each of the four regions of $(0,1)^3$ given by the $p1 = p2$ and $p1 = p3$ planes (**A**, **B**, **C**, **D**).

and $p_1 < p_3$. Consider any $B_1 = (p_1, p_2, p_3)$ in region **B**. By the intermediate value theorem, if in B_1, unlike B, $p_4^{*RD} > p_4^{*RR^*}$ or $p_4^{*RD} < p_4^{*RR}$, then for all continuous paths from B to B_1, even those living entirely inside region **B**, there must exist a point $(p_1, p_2 < p_1, p_3 > p_1)$ at which $p_4^{*RD} = p_4^{*RR^*}$ or $p_4^{*RD} = p_4^{*RR}$, respectively. But by Lemma, there are no points in region **B**, or **A** or **C** or **D**, for which $p_4^{*RD} = p_4^{*RR^*}$ or $p_4^{*RD} = p_4^{*RR}$. Thus no region has any point (p_1, p_2, p_3) contradicting the $p_4^{*RR} < p_4^{*RD} < p_4^{*RR^*}$ or $p_4^{*RR} > p_4^{*RD} > p_4^{*RR^*}$ character shown in that region's example, so the theorem holds for all points in each region. In the remaining cases, $p_1 = p_2$ or $p_1 = p_3$, so all effect measures agree intuitively from the relationship between p_3 and p_4, or p_2 and p_4, respectively. All $(p_1, p_2, p_3) \in (0,1)^3$ lie in one of the four regions or the $p_1 = p_2$ or $p_1 = p_3$ plane, so if $p_4 \in (0,1)$ enables agreement between RR and RR*, then RD will also agree as desired.

Lemma: ($p_4^{*RD} = p_4^{*RR^*}$ or $p_4^{*RD} = p_4^{*RR}$) implies ($p_1 = p_2$ or $p_1 = p_3$)

Proof: Algebra confirms this Lemma for $p_4^{*RD} = p_4^{*RR}$:

$$p_2 + p_3 - p_1 = \frac{p_2 p_3}{p_1}$$

$$p_1 p_2 + p_1 p_3 - p_1^2 - p_2 p_3 = 0$$

$$(p_1 - p_2)(p_1 - p_3) = 0$$

$$p_1 = p_2 \text{ or } p_1 = p_3$$

And similarly for $p_4^{*RD} = p_4^{*RR^*}$:

$$p_2 + p_3 - p_1 = 1 - \frac{(1-p_2)(1-p_3)}{1-p_1}$$
$$(1-p_1)(p_2+p_3-p_1) = 1 - p_1 - (1-p_2)(1-p_3)$$
$$p_1^2 - (p_2+p_3+1)p_1 + p_2 + p_3 = -p_1 + p_2 + p_3 - p_2p_3$$
$$p_1^2 - (p_2+p_3)p_1 + p_2p_3 = 0$$
$$(p_1-p_2)(p_1-p_3) = 0$$
$$p_1 = p_2 \text{ or } p_1 = p_3$$

Therefore, if $p_4^{*RD} = p_4^{*RR^*}$ or $p_4^{*RD} = p_4^{*RR}$, then $p_1 = p_2$ or $p_1 = p_3$ as desired.

Theorem: Let $p1, p2, p3, p4$ be independent random variables each following a uniform (0,1) distribution. The probability that the set of effect measures {RR, RR*, HR, HR*, RD, OR} agrees is 5/6.

Proof: By our theorem in Supplement A, it suffices to show that the probability of RR and RR* disagreeing is $\frac{1}{6}$. For each point in $(0,1)^3$, the conditional probability that RR and RR* disagree is the probability that $p4$ falls between $p4^{*RR}$ and $p4^{*RR^*}$. Since $p4$ takes a $(0,1)$ uniform distribution, this probability is $\min\{1, \max\{p4^{*RR}, p4^{*RR^*}\}\} - \max\{0, \min\{p4^{*RR}, p4^{*RR^*}\}\}$. Hence the overall probability P that RR and RR* disagree is

$$\iiint_{(0,1)^3} (\min\{1, \max\{p4^{*RR}, p4^{*RR^*}\}\} - \max\{0, \min\{p4^{*RR}, p4^{*RR^*}\}\}) dp_{1,2,3}.$$

We partition $(0,1)^3$ into regions **A**, **B**, **C**, and **D** as above. This gives $P =$

$$\iiint_{\mathbf{A}} (\min\{1, \max\{p4^{*RR}, p4^{*RR^*}\}\} - \max\{0, \min\{p4^{*RR}, p4^{*RR^*}\}\})\, dp_{1,2,3}$$
$$+ \iiint_{\mathbf{B}} (\min\{1, \max\{p4^{*RR}, p4^{*RR^*}\}\} - \max\{0, \min\{p4^{*RR}, p4^{*RR^*}\}\}) dp_{1,2,3}$$
$$+ \iiint_{\mathbf{C}} (\min\{1, \max\{p4^{*RR}, p4^{*RR^*}\}\} - \max\{0, \min\{p4^{*RR}, p4^{*RR^*}\}\}) dp_{1,2,3}$$
$$+ \iiint_{\mathbf{D}} (\min\{1, \max\{p4^{*RR}, p4^{*RR^*}\}\} - \max\{0, \min\{p4^{*RR}, p4^{*RR^*}\}\}) dp_{1,2,3}$$

Each of these integrals evaluates to $\frac{1}{24}$. We will compute the first integral; we leave the rest to the reader.

We know that the following are always true in region **A**:

- $p1 < p2$ (by definition of region **A**)
- $p1 < p3$ (by definition of region **A**)
- $p4^{*RR} > p4^{*RR^*} \geq 0$. Earlier, we used the intermediate value theorem to show that the above imply $p4^{*RR} > p4^{*RD} > p4^{*RR^*}$. Furthermore, $p4^{*RR^*} < 0$ would imply $p3 < 1 - \frac{1-p1}{1-p2}$, an impossibility in region **A** since $p1 < p2$.

Hence we can resolve the extrema in our integral:

$$\iiint_{\mathbf{A}} (\min\{1, \max\{p4^{*RR}, p4^{*RR^*}\}\} - \max\{0, \min\{p4^{*RR}, p4^{*RR^*}\}\})\, dp_{1,2,3}$$
$$= \iiint_{\mathbf{A}} (\min\{1, p4^{*RR}\} - p4^{*RR^*})\, dp_{1,2,3}$$

To resolve the remaining minimum, we will separately integrate the subregions in which each candidate is the minimum:

$$\int_0^1 \int_{p1}^1 \int_{p1}^1 (\min\{1, \frac{p2p3}{p1}\} - (1 - \frac{(1-p2)(1-p3)}{1-p1}))\, dp3\, dp2\, dp1$$
$$= \int_0^1 \int_{p1}^1 \int_{p1}^{\frac{p1}{p2}} \frac{p2p3}{p1}\, dp3\, dp2\, dp1$$
$$+ \int_0^1 \int_{p1}^1 \int_{\frac{p1}{p2}}^1 1\, dp3\, dp2\, dp1$$
$$- \int_0^1 \int_{p1}^1 \int_{p1}^1 (1 - \frac{(1-p2)(1-p3)}{1-p1})\, dp3\, dp2\, dp1$$
$$= \frac{1}{16} + \frac{1}{4} - \frac{13}{48} = \frac{1}{24}$$

Our integration over region **A** shows that the probability that $p1 < p2$, $p1 < p3$, and the two relative risks disagree is $\frac{1}{24}$. Similar integration over regions **B**, **C**, and **D** shows that probability to be $\frac{1}{24}$ for each of the other three inequality cases. Hence the overall probability that the two relative risks disagree is $\frac{1}{6}$. Applying our Theorem from Supplement A, we see that all our effect measures {RR, RR*, HR, HR*, RD, OR} agree with probability $\frac{5}{6}$ as desired.

C Supplement: Proof of Inequalities

In this supplement, we show that if $0 < p4 < p2 < 1 < \frac{1-p1}{1-p2} < \frac{1-p3}{1-p4}$, then $\frac{\log(1-\tilde{p_2}\mathrm{RR}^*_P)}{\log(1-\tilde{p_2})} < \frac{\log(1-\tilde{p_4}\mathrm{RR}^*_P)}{\log(1-\tilde{p_4})} < \frac{\log(1-\tilde{p_4}\mathrm{RR}^*_Q)}{\log(1-\tilde{p_4})}$.

Proof of $\frac{\log(1-\tilde{p_4}\mathbf{RR^*}_P)}{\log(1-\tilde{p_4})} < \frac{\log(1-\tilde{p_4}\mathbf{RR^*}_Q)}{\log(1-\tilde{p_4})}$

By assumption, $\mathrm{RR^*_P} < \mathrm{RR^*_Q}$. Equivalently, $\log(1-\tilde{p_4}\mathrm{RR}^*_P) > \log(1-\tilde{p_4}\mathrm{RR}^*_Q)$. We now divide both sides by the negative value $\log p4 = \log(1-\tilde{p_4})$, giving $\frac{\log(1-\tilde{p_4}\mathrm{RR}^*_P)}{\log(1-\tilde{p_4})} < \frac{\log(1-\tilde{p_4}\mathrm{RR}^*_Q)}{\log(1-\tilde{p_4})}$ as desired.

Proof of $\frac{\log(1-\tilde{p_2}\mathbf{RR^*}_P)}{\log(1-\tilde{p_2})} < \frac{\log(1-\tilde{p_4}\mathbf{RR^*}_P)}{\log(1-\tilde{p_4})}$

Let x be such that $\tilde{p_2} \leq x \leq \tilde{p_4}$. Since $0 < x < 1$, we have $-\frac{x^2}{2} - \frac{x^3}{3} - \ldots < 0$. Equivalently, $-x - \frac{x^2}{2} - \frac{x^3}{3} - \ldots < -x = \frac{x^2-x}{1-x}$. The left side is the power series for $\log(1-x)$, so $\log(1-x) < \frac{x^2-x}{1-x}$ and $x + (1-x)\log(1-x) < x^2$. Rearranging, $-\frac{x}{1-x} - \log(1-x) > -\frac{x^2}{1-x}$ and $\frac{\frac{-x}{1-x} - \log(1-x)}{x^2} + \frac{1}{1-x} > 0$. But the left side is the derivative of $\frac{\log(1-x)}{x} - \log(1-x)$, so that expression must be strictly increasing over the open unit interval (as $0 < x < 1$ was the only property of x we used so far). In particular, $x < x\mathrm{RR}^*_P = \frac{x\tilde{p_1}}{\tilde{p_2}} \leq \frac{\tilde{p_4}\tilde{p_1}}{\tilde{p_2}} < \tilde{p_3} < 1$, so $\frac{1-x}{x}\log(1-x) < \frac{1-x\mathrm{RR}^*_P}{x\mathrm{RR}^*_P}\log(1-x\mathrm{RR}^*_P)$. Rearranging, $\frac{(1-x\mathrm{RR}^*_P)\log(1-x\mathrm{RR}^*_P)}{(1-x)\log(1-x)} < \mathrm{RR}^*_P$ and $\frac{\log(1-x\mathrm{RR}^*_P)}{1-x} - \frac{\mathrm{RR}^*_P \log(1-x)}{1-x\mathrm{RR}^*_P} > 0$. Dividing both sides by the positive $(\log(1-x))^2$, the left side becomes the derivative of $\frac{\log(1-x\mathrm{RR}^*_P)}{\log(1-x)}$. Hence that expression is strictly increasing over $\tilde{p_2} \leq x \leq \tilde{p_4}$. As desired, $\frac{\log(1-\tilde{p_2}\mathrm{RR}^*_P)}{\log(1-\tilde{p_2})} < \frac{\log(1-\tilde{p_4}\mathrm{RR}^*_P)}{\log(1-\tilde{p_4})}$.

D Supplement: Remedial Measure for Cumulative Hazard Ratios

In this supplement, we provide a remedial measure for defining the cumulative hazard ratios when the equal follow-up periods assumption fails.

If the assumption of equal follow-up periods does not hold, it may be possible to define the cumulative hazard ratios by letting t_f represent the lesser of the durations of the follow-up periods. If the proportional hazards (recovery rates) assumption holds, then the interpretation of HR (HR*) seen in our case studies holds during the shorter follow-up period. However, HR (HR*) would no longer consider all participants reaching (recovering from) the outcome, so we would no longer have HR $= \frac{\log(1-p2)}{\log(1-p1)}$ (HR* $= \frac{\log p1}{\log p2}$) in general. It would then be possible for HR (HR*) to disagree with other effect measures even when RR and RR* agree.
